# Asthma prevalence, lung and cardiovascular function in adolescents born preterm

**DOI:** 10.1038/s41598-020-76614-0

**Published:** 2020-11-12

**Authors:** Maria Arroyas, Cristina Calvo, Santiago Rueda, Maria Esquivias, Cristina Gonzalez-Menchen, Ersilia Gonzalez-Carrasco, Maria Luz Garcia-Garcia

**Affiliations:** 1 Pediatrics Department, Severo Ochoa University Hospital, Avenida Orellana S/N. Leganes, 28911 Madrid, Spain; 2 Alfonso X El Sabio University, Villanueva de la Cañada, Madrid, Spain; 3 Pediatrics Department, La Paz University Hospital, Madrid, Spain; 4 Translational Research Network in Pediatric Infectious Diseases (RITIP), Madrid, Spain; 5 TEDDY Network (European Network of Excellence for Pediatric Clinical Research), Bari, Italy; 6 Pediatrics Department, Clinico San Carlos University Hospital, Madrid, Spain

**Keywords:** Cardiology, Medical research

## Abstract

Our main objective was to study respiratory evolution and pulmonary and cardiac function in adolescents born preterm in the post-surfactant era. Observational cross-sectional study, comparing very preterm (< 32 weeks) and moderately-late preterm adolescents (≥ 32 weeks) (74 each group). We recorded respiratory symptoms, spirometry and functional echocardiogram. Very preterm adolescents required more respiratory admissions (45.9% vs. 28.4%) (*p* = 0.03, OR 2.1, CI95% 1.1–4.2) and had more current asthma (21.6% vs. 9.5%, *p* = 0.04, OR 2.3, CI95% 1.1–5.2). Preterm subjects with intrauterine growth restriction (IUGR) presented lower FEV_1_ (88.7 ± 13.9 vs. 95.9 ± 13.3, *p* = 0.027) and lower FVC (88.2 ± 13.6 vs. 95.5 ± 13.3, *p* = 0.025). When assessing right ventricle, very preterm showed a greater E/E’ ratio (*p* = 0.02) and longer myocardial performance index (MPI) (*p* = 0.001). Adolescents with IUGR showed less shortening fraction (*p* = 0.016), worse E/E′ ratio (*p* = 0.029) and longer MPI (*p* = 0.06). Regarding left ventricle, very preterm showed less E′ wave velocity (*p* = 0.03), greater E/E′ ratio (*p* = 0.005) and longer MPI (*p* < 0.001). Gestational age < 32 weeks is independently associated with current asthma in adolescence. Children 13–14 years old born very preterm required more respiratory admissions and had poorer diastolic and global function of both ventricles. IUGR is a risk factor for poorer lung function in preterm adolescents, regardless gestational age.

## Introduction

Preterm birth represents a growing problem across the world with a global incidence of 15 million per year^1^. Treatment strategies have improved in the last decades, resulting in an increased survival of the most immature babies. However, these patients are at risk of developing sequelae, not only in the neonatal period, but also in preschool age, adolescence and adult life. Very and extremely preterm babies are the most likely to be affected as they were born at such a vulnerable period. Nevertheless, moderately-late preterm constitute the main group of preterm babies nowadays and recent evidence suggests more long-term morbidities than term-born babies^2,3^.

Prematurity has been associated with a poorer vascular growth, endothelial disfunction and less nitric oxide and elastin production^4^, resulting in arterial stiffness that may explain the risk of hypertension (HT) seen in preterm adults, regardless of genetic or environmental factors^5^. The association of HT with other risk factors also observed in preterm adults such as dyslipidaemia or insulin resistance^6^, result in an increased susceptibility to develop ischemic cardiopathy and cerebrovascular accident^7^. It has also been described in preterm patients an increased pulmonary vascular reactivity, resulting in cardiac remodelling leading to changes in structure and geometry of right ventricle^8^, associated with systolic and diastolic dysfunction^9,10^.

In addition to cardiovascular consequences, preterm birth affects airway growth and lung development in a way that may contribute to long-term respiratory morbidities^11,12^. Not only preterm birth is a risk factor for poor lung development, but also bronchopulmonary dysplasia (BPD) and intrauterine growth restriction (IUGR)^13^. In preschool children born preterm, it has been described an obstructive pattern with a decreased forced expiratory volume in one second (FEV_1_), more wheezing episodes and more frequent hospital admissions^14^. At school age, preterm birth has been associated with a decreased FEV_1_, forced vital capacity (FVC) and mean forced expiratory flow between 25 and 75% of FVC (FEF_25-75_)^15^. This obstructive pattern seems to persist in young adults, associated with more wheezing episodes and more need of treatment to control respiratory symptoms^16^.

However, it is important to bear in mind that most publications about the effects of preterm birth in adolescents and young adults include patients born before the spread of surfactant therapy, antenatal corticosteroids and more gentle ventilation techniques in Neonatal Units^17,18^. These new strategies have raised the survival rate of the most immature infants and probably have contributed to a better long-term functional situation of patients born preterm. However, although the follow-up of this “new” preterm children in the first years of life is well established, it is necessary to improve our knowledge of medium and long-term consequences of prematurity, once they become adolescents and adults. 

The main aim of this study was to describe the clinical, respiratory and cardiovascular situation of 13–14-year adolescents born preterm in the post surfactant era, comparing extremely and very preterm with moderately-late preterm.

## Methods

An observational analytic cross-sectional study was performed, including babies born in 2003 and 2004 in the Severo Ochoa Hospital (Leganés, Spain) and Clínico San Carlos Hospital (Madrid, Spain). All patients born below 37 weeks were included, except for infants with congenital defects and neurological disability. Finally, 148 of them agreed to undergo the study. Children were recruited at mean age of 13–14 years. The study population consisted of 74 adolescents born below 32^0^ weeks of gestation (very preterm group) and the comparison group of 74 age-matched adolescents born between 32^1^ and 36^6^ weeks (moderately-late preterm group). In addition, very preterm adolescents with BPD (n = 21) were compared with very preterm with no BPD (n = 53). All adolescents born below 37 weeks with IUGR (n = 20) were compared with those without IUGR (n = 128).

The study was approved by the Ethics Committee of Severo Ochoa Hospital. Written informed consent was obtained from all the adolescents and their parent/caregiver after full explanation of the study protocol. All methods were carried out in accordance with relevant guidelines and regulations and was conducted in accordance with the Declaration of Helsinki.

The follow-up included anthropometric measurements (weight, height, body mass index-BMI presented with z-scores WHO 2006–2007). Perinatal data were collected from medical history (newborn weight was presented with Fenton z-scores^19^).

Intrauterine growth restriction was defined as fetal weight below 3 percentile or below 10 percentile and altered doppler of uterine, cerebral or umbilical arteries^20^. BPD in very preterm infants was defined as need of supplementary oxygen at 28 days of life^21^.

The International Study of Asthma and Allergies in Childhood (ISAAC) questionnaire for asthma symptoms for adolescents 13–14 years, previously validated and translated to Spanish, was answered by patients^22^. Current prevalence of asthma was estimated by the percentage of patients with an affirmative answer to question number 2 (*have you had wheezing episodes over the last 12 months?*), which has previously shown the higher correlation with prevalence of asthma in validation studies^23^.

### Blood pressure

Blood pressure was measured oscillometrically (Phillips Sure Signs VS3, MA, USA) three times taking final values averaged from three readings. Results were presented with z-scores (National High Blood Pressure Education Group on High Blood Pressure in Children and Adolescents^24^).

### Lung function

Spirometry was performed according to established guidelines^25^ using a Jaeger MasterScope-PC spirometer (VIASYS HealthCare GmbH, Hoechberg, Germany). At least three reproducible manoeuvres were performed, selecting the one with best FEV-_1_ and FVC values. The following spirometry values were collected: FVC, FEV_1_, FEV_1_/FVC and FEF_25–75_. Results were presented as percentage of predicted values with reference values of Zapletal^26^ and z-score of predicted values with reference values of the Global Lung Function Initiative (GLI)^27^. The bronchodilator test was considered positive when there was a 12% increase in the FEV_1_ compared with baseline after administration of 400 μg salbutamol.

### Exhaled fraction of nitric oxide (FeNO)

FeNO was determined using the portable NIOX VERO (R) device, considering normal FeNO values ​​ < 25 ppb^28^.

### Skin prick tests

Allergic sensitization was evaluated performing skin prick tests (SPT) for common inhaled allergens. Standardized extracts (Abelló) were used with a positive control (10 mg/ml histamine) and a negative control (glycerol-saline carrier solution). Papule diameters equal to or exceeding that obtained with histamine were considered positive.

### Echocardiography

Transthoracic echocardiography was performed using Vivid Pro 7 sonography (General Electric Healthcare, USA), using a 3-MHz probe, by three experienced paediatric echocardiographers using European Society of Cardiology (ESC) standard guidelines. Right atrial tricuspid blood regurgitation jet was used to evaluate systolic pulmonary artery pressure, assuming the right atrial pressure to be 5 mmHg. M-mode recordings were used to measure cardiac dimensions, power-doppler and continuous-doppler to measure velocities and tissue doppler for myocardial performance index (MPI). Results were presented with z-scores^29–31^.

### Statistical analysis

To calculate the sample size, the prevalence of asthma in adolescents born very preterm was expected to be around 25–30%^32^, whereas in the moderately-late preterm was expected to be similar to general population (10%)^33^. The minimum sample size (80% power, alfa error 0.05), required was 60 patients in each group.

Absolute and relative frequencies (%) were used to describe qualitative variables, whereas quantitative ones were expressed as media and standard deviation, in case of Normal distribution, or median and interquartile range for variables not normally distributed. Comparisons were performed by *X*^2^*,* Student’s t test and Mann–Whitney test.

To control for potentially confounding variables and to examine the independent contribution of the explicative variables on the likelihood of developing asthma, a backward stepwise binomial logistic regression model was built. All the variables with P-value < 0.1 were introduced in the multi-variate analysis. Adjusted odds ratios (OR) with 95% confidence intervals were calculated. To study the risk factors predicting lung function among children born preterm, we established a general linear model.

All analyses were performed using the Statistical Package for the Social Sciences (SPSS), Version 21.0.

## Results

148 patients with mean age 14.1 years (15.3–12.7) were included (74 very preterm and 74 moderately-late preterm). Very preterm children were slightly younger (13.9, IQR 14.4–13.5 years vs. 14.3, IQR 14.5–14.1 years, *p* < 0.001). There was no difference in gender between groups. IUGR was more prevalent in ≥ 32 wGA (*p* = 0.05) and in females (19% *vs*. 8%, *p* = 0.05). Perinatal data are presented in Table 1.

**Table 1:** Perinatal characteristics of very preterm and moderately-late preterm.

	< 32 weeks N = 74	≥ 32 weeks N = 74	Odds Ratio (CI 95%)	*P* value
Male gender	36 (48.6%)	38 (51.4%)	0.9 (0.7–1.3)	0.869
Multiple pregnancy	22 (29.7%)	22 (29.7%)	1 (0.6–1.6)	1
Intrauterine growth restriction	6 (8.1%)	14 (18.7%)	0.43 (0.17–1)	0.050
Preeclampsia	16 (14.9%)	13 (17.6%)	0.8 (0.42–1.6	0.500
Maternal smoking in pregnancy	11 (14.9%)	17 (23%)	0.6 (0.3–1.2)	0.200
Maternal age > 35 years	24 (32.4%)	16 (21.6%)	1.5 (0.8–2.6)	0.140
Gestational age (weeks)*	28.0 ± 1.9	34.2 ± 1.2		< 0.001
Newborn weight (g)	1096 ± 363	2041 ± 526		< 0.001
z-score**	− 0.11 ± 1.25	− 0.66 ± 1.21		0.006
BPD	21	0		< 0.001
Surfactant therapy	41 (55.4%)	2 (2.7%)	0.05 (0.01–0.19)	< 0.001
Patent ductus arteriosus	19 (25.7%)	0	1.35 (1.2–1.5)	< 0.001
Noninvasive mechanical ventilation (NIMV)	57 (77%)	5 (6.8%)	11.4 (4.8–20.2)	< 0.001
Duration NIMV	6.8 ± 9.7	1.2 ± 0.7		0.070
Invasive mechanical ventilation	48 (68.9%)	3 (4.1%)		< 0.001

*Mean ± standard deviation.

**Z-score Fenton 2013.

### Anthropometric data

Anthropometric data are shown in Table 1S. Weight and z-score at follow-up were significantly lower in the very preterm group compared to moderately-late preterm (*p* < 0.001 and *p* = 0.005 respectively). Height was also lower in the very preterm group (*p* = 0.007), but no difference was found in its z-score. BMI and its z-score were also lower in the very preterm group compared to moderately-late preterm (*p* = 0.001 and *p* = 0.004).

However, no differences were found when comparing preterm adolescents with and without IUGR in weight, height, BMI and its z-scores.

### Respiratory health

The risk of hospital admission due to respiratory problems throughout childhood was 4.5 times higher in < 32 wGA (*p* = 0.03) and 2.2 times higher when the admission was in ICU (*p* = 0.03). The very preterm group also needed more frequently inhaled corticosteroids throughout childhood to control asthma symptoms (*p* < 0.001). No differences were found in the frequency of allergic sensitization and parental asthma/atopy as presented in Table 2.

**Table 2:** Respiratory evolution of the Study Groups at Follow-Up according to gestational age.

	< 32 weeks N = 74	≥ 32 weeks N = 74	Odds ratio Confidence Interval 95%	*P* value
Respiratory admissions	34 (46%)	21 (28%)	2.1 (1.1–4.2)	0.030
Respiratory ICU admissions	9 (12%)	2 (2.7%)	4.5 (1.1–20.1)	0.030
Montelukast	58 (78%)	57 (77%)	1.1 (0.5–2.3)	0.843
Inhaled corticosteroids	35 (47%)	16 (22%)	3.3 (1.6–6.7)	0.001
Allergic sensitization	21 (54%)	27 (40%)	1.7 (0.8–2.3)	0.177
Parental asthma	16(12%)	16 (12%)	1.0 (0.4–2.4)	1
Parental atopy	30 (40.5%)	23 (31%)	1.5 (0.8–2.9)	0.230

Answers to the ISAAC questionnaire for asthma symptoms are presented in Table 2S. The frequency of current asthma was 21.6% in < 32 wGA vs. 9.5% in ≥ 32 wGA, *p* = 0.04, OR: 2.3; CI95% 1.1–5.2. Ever asthma and nocturnal cough were also significantly more prevalent in the very preterm group compared to the moderately-late preterm one.

Besides gestational age < 32 wGA, other factors also associated with current asthma were: parental asthma (29.2% vs. 13%, *p* = 0.04, OR 2.8; CI 95% 1.1–7.7) and allergic sensitization (23% vs. 7%, *p* = 0.02, OR 4.0; CI 95% 1.2–13.6). BPD was also more frequent in adolescents with current asthma, although the difference was not statistically significant (*p* = 0.070).

After logistic regression, the variables independently associated with current asthma at 13–14 years were: allergic sensitization (*p* = 0.02, OR 4.1; CI95%:1.2–14.1), paternal asthma (*p* = 0.04, OR 2.9; CI95%:1.1–8.2) and gestational age < 32 (*p* = 0.04, OR 2.9; CI95%:1.1–7.2).

### Exhaled nitric oxide and allergic sensitization

Although levels of exhaled nitric oxide were within normal range in both groups, median values were lower in the very preterm group (12.5, IQR 12 ppb) than in the moderately-late preterm group (15.5, RIQ 20,2 ppb) (*p* = 0.02).

### Lung function

The mean spirometry measurements obtained in both, very preterm and moderately-late preterm adolescents, were normal and no differences between groups were found. Data are presented in Table 3S. When extremely preterm adolescents (< 28wGA) were selected, FEF_25-75_ and its z-score were significantly lower compared to moderately-late preterm (*p* = 0.02, *p* = 0.04 respectively).

**Table 3:** Predicted influence of gender, IUGR and gestational age < 32 weeks on lung function at adolescence among all participating children.

		Regression coefficient	95% CI of regression coefficient	P value	R^2^	Adjusted R^2^
FEV-_1_	GA < 32 weeks	0.02	− 3.5 to 4.9	0.757	0.130	0.112
	IUGR	0.14	− 0.9 to 11.6	0.070		
	Female	− 0.34	− 12.7 to − 4.3	< 0.001		
FVC	GA < 32 weeks	0.03	− 3.5 to 4.9	0.736	0.110	0.092
	IUGR	0.1	− 5.9 to 12.0	0.080		
	Female	− 0.3	− 11.7 to − 3.2	0.001		
FEF _25–75_	GA < 32 weeks	0.2	− 0.1 to 14.7	0.050	0.085	0.066
	IUGR	0.1	− 4.9 to 17.1	0.278		
	Female	− 0.2	− 17.7 to − 2.9	0.007		

No differences were found in lung function values among very preterm adolescents with and without BPD. However, when the whole group of preterm adolescents with and without IUGR were compared, those with IUGR presented lower FEV_1_ (*p* = 0.03), lower FEV_1_ z-score (*p* = 0.03), lower FVC (*p* = 0.02) and lower FVC z-score (*p* = 0.03).

To study the risk factors predicting lung function among children born preterm, we established a general linear model that is shown in the Table 3. The variables significantly associated with lung function in the bivariate analysis: IUGR and male gender were considered. Also, gestational age < 32 wGA, as one of the main variables in the study, was included. Using FEV_1_ (expressed as % predicted value) as the dependent variable and gender, GA < 32 wGA and IUGR as predictors, the model was statistically significant and accounted for 11.2% of the variance in FEV_1_ (*p* < 0.001). IUGR associated with reduced FEV_1_ and FVC, although the difference did not reach statistical significance (*p* = 0.07 and *p* = 0.08 respectively). Prematurity < 32 wGA, adjusted with female gender and IUGR, was significant in MEF50, but not in FEV-1 or FVC.

### Blood pressure

Blood pressure results of the study populations are shown in Table 4. Systolic blood pressure (SBP) and z-score were similar in very preterm and moderately-late preterm. However, diastolic blood pressure (DBP) and its z-score were higher in very preterm adolescents compared to controls (*p* = 0.040, *p* = 0.003 respectively). When extremely-preterm born (< 28^0^ weeks) were selected, both SBP, DBP and their z-score were higher compared to moderately-late preterm group.

**Table 4:** Blood pressure at adolescence according gestational age and the occurrence of intrauterine growth restriction (IUGR).

	< 32 weeks N = 74	≥ 32 weeksN = 74	*P* value
Systolic blood pressure*	110 (19.8)	110 (20)	0.510
Systolic blood pressure z-score*	0.34 (1.3)	0.34 (1.3)	0.178
Diastolic blood pressure*	62 (10)	60 (8.8)	0.040
Diastolic blood pressure z-score*	0.08 (1)	− 0.36 (0.73)	0.003

	< 28 weeks N = 37	≥ 32 weeks N = 74	*P* value
Systolic blood pressure*	114 (27.5)	110 (20)	0.040
Systolic blood pressure z-score*	0.54 (1.9)	0.34 (1.3)	0.050
Diastolic blood pressure*	63 (17)	60 (5)	0.030
Diastolic blood pressure z-score*	0.14 (1)	− 0.36 (0,73)	0.020

	IUGR N = 20	Non-IUGR N = 128	*P* value
Systolic blood pressure*	110 (10)	110 (19)	0.124
Systolic blood pressure z-score*	− 0.13 (1.42)	0.07 (1.31)	0.292
Diastolic blood pressure*	70 (14)	60 (10)	0.230
Diastolic blood pressure z-score*	− 0.31 (0.79)	− 0.24 (0.98)	0.186

*Median (Interquartile range).

No significant differences in systolic or diastolic blood pressure were found between IUGR and non-IUGR groups.

### Cardiac function

Data obtained in the assessment of right ventricle in very preterm and moderately-late preterm are shown in Table 5. There was a trend toward a smaller right ventricle in the very preterm group (basal diameter z-score). No difference was found in systolic function (TAPSE, shortening fraction, S′ velocity). Nonetheless, data suggesting worse right ventricle diastolic function were found in very preterm infants, who showed a greater E/E’ (p = 0.017) and worse global right ventricle function determined by MPI (*p* < 0.001).

**Table 5:** Values of right ventricle’s function obtained by echocardiography at adolescence according gestational age and the occurrence of intrauterine growth restriction (IUGR).

	< 32 weeks N = 74	≥ 32 weeks N = 74	*P* value	IUGR N = 20	Non-IUGR N = 128	*P* value
Length (mm)*	72.2 ± 2	65.9 ± 11	0.17	64 ± 8.5	65 ± 9.0	0.600
Basal diameter (mm)*	31.2 ± 5.9	33.5 ± 5.0	0.016	30.6 ± 3.7	32.8 ± 5.8	0.016
Z-score basal diameter*	1.6 ± 0.9	1.7 ± 0.8	0.17	1.5 ± 0.8	1.6 ±	0.240
TAPSE (mm)*	24.1 ± 5.0	23.2 ± 3.4	0.59	22.5 ± 3.9	23.9 ± 4.3	0.170
Shortening fraction (%)*	38.7 ± 7.6	39.9 ± 8.15	0.37	38.6 ± 7.9	43.2 ± 1.01	0.016
S’ wave (cm/s)*	12.6 ± 2.3	13.3 ± 2.5	0.09	13 ± 2.1	13 ± 2.5	0.990
E/A ratio*	1.4 ± 0.2	1.7 ± 0.6	0.48	1.8 ± 0.34	1.7 ± 0.37	0.330
E’ wave (cm/s)*	39 ± 19	49 ± 29	0.04	16.3 ± 20	17 ± 26	0.580
E’ wave z-score*	− 0.53 ± 1.10	− 0.19 ± 1.17	0.082	− 0.15 ± 0.71	− 0.29 ± 1.2	0.370
E/E’ ratio*	3.9 ± 1.5	3.3 ± 1.02	0.017	3.7 ± 1.3	3 ± 0.9	0.029
E/E’ ratio z-score*	0.2 ± 1.3	− 0.3 ± 0.9	0.006	0.33 ± 0.95	− 0.03 ± 1.2	0.030
Myocardial performance index (MPI)*	0.39 ± 0.13	0.32 ± 0.08	< 0.001	0.36 ± 0.11	0.31 ± 0.09	0.060
MPI z-score*	0.61 ± 1.1	− 0.6 ± 0.72	0.01	0.59 ± 0.85	− 0.38 ± 0.94	0.090

*Mean ± standard deviation.

TAPSE, Tricuspid annular plane systolic excursion; MPI, myocardial performance index.

The assessment of right ventricle in preterm adolescents with IUGR showed worse systolic and diastolic function compared to no-IUGR ones, with less shortening fraction (p = 0.016), worse E/E’ ratio (*p* = 0.03) and longer MPI (*p* = 0.06).

Systolic pulmonary artery pressure (SPAP) was estimated in 40 of 148 adolescents (27%) and was higher in those born very preterm (24.9 ± 5.6 mmHg) compared to moderately-late preterm (20.9 ± 4.2 mmHg) (*p* = 0.04). No difference was found when comparing preterm adolescents with or without BPD or IUGR.

Data obtained in the assessment of left ventricle are showed in Table 6. A smaller diastolic diameter was observed diameter in the very preterm compared to moderately -late preterm (*p* = 0.04). No differences in systolic function were found. However, diastolic function seemed to be lower in very preterm adolescents, as shown by lower E′ wave velocity and its z-score (*p* = 0.015 and *p* = 0.02 respectively) and lower E/E′ ratio (*p* = 0.05) and E/E′ ratio z-score (*p* = 0.02). Global function showed a greater septal MPI and its z-score in very preterm compared to moderately-late preterm (*p* < 0.001 and *p* = 0.01 respectively).

**Table 6:** Values of left ventricle’s morphology and function obtained by echocardiography in very preterm and moderately-late preterm adolescents.

	< 32 weeks N = 74	≥ 32 weeks N = 74	*P* value	IUGR N = 20	Non-IUGR N = 128	*P* value
Left ventricle diastolic diameter (mm)*	44.0 ± 4.2	45.4 ± 4.3	*p* = 0.040	44.6 ± 4.9	44.7 ± 4.2	0.920
Left ventricle diastolic diameter z-score*	− 0.29 ± 1.26	− 0.21 ± 1	*p* = 0.070	− 0.38 ± 1.5	− 0.23 ± 1.08	0.590
Interventricular septum (diastole) (mm)*	6.9 ± 1.2	7.3 ± 1.2	*p* = 0.070	6.9 ± 1.2	7.2 ± 1.2	0.410
Interventricular septum z-score*	− 0.13 ± 0.76	0.10 ± 0.78	*p* = 0.770	− 0.23 ± 0.65	− 0.1 ± 0.78	0.470
Left posterior wall (diastole) (mm)*	7.2 ± 1,7	7.36 ± 1.2	*p* = 0.029	7.12 ± 1.2	7.4 ± 1.2	0.290
Left posterior wall z z-score*	0.2 ± 0.9	0.3 ± 0.5	*p* = 0.700	0.41 ± 0.27	0.83 ± 0.94	0.480
Shortening fraction (%)	38.8 ± 4.3	39.4 ± 4.8	*p* = 0.270	38.2 ± 4.2	39.1 ± 4.6	0.430
Ejection fraction (%)	68.8 ± 5.6	69.9 ± 5.5	*p* = 0.230	68.5 ± 5.3	69.5 ± 5.5	0.650
S’ wave velocity (cm/s)*	11.4 ± 3.1	12.4 ± 1.4	*p* = 0.020	10.8 ± 3.2	13.5 ± 1.06	0.120
S’ wave velocity z-score*	− 0.28 ± 1.3	− 0.1 ± 1.04	*p* = 0.360	− 0.44 ± 1.2	− 0.02 ± 1.1	0.650
E’ wave velocity (cm/s)*	18.5 ± 3.5	21.5 ± 2.7	*p* = 0.015	17.5 ± 2.04	19.7 ± 0.3	0.690
E’ wave velocity z-score*	− 0.47 ± 1.04	− 0.06 ± 0.8	*p* = 0.020	− 0.14 ± 1.04	− 0.27 ± 0.95	0.570
E/E’ ratio*	5.2 ± 1.1	4.2 ± 1.1	*p* = 0.050	4.5 ± 1.4	4.3 ± 1.0	0.710
E/E’ ratio z-score*	0.66 ± 0.79	− 0.35 ± 0.83	*p* = 0.020	− 0.31 ± 0.99	− 0.23 ± 0.82	0.760
Septal MPI*	0.37 ± 0.09	0.29 ± 0.05	*p* < 0.001	0.32 ± 0.06	0.32 ± 0.08	0.560
Septal MPI z-score*	0.32 ± 0.85	0.10 ± 1.02	*p* = 0.010	− 0.7 ± 0.84	− 0.74 ± 0.78	0.830
Lateral MPI*	0.32 ± 0.09	0.28 ± 0.05	*p* = 0.001	0.30 ± 0.08	0.30 ± 0.06	1
Lateral MPI z-score*	0.19 ± 0.86	0.11 ± 0.76	*p* = 0.500	− 0.7 ± 0.71	− 0.97 ± 0.7	0.140

Mean ± standard deviation.

MPI, myocardial performance index.

No differences were found when comparing adolescents with and without BPD. However, when very preterm adolescents with moderate-severe BPD were compared to very preterm ones without BPD, the first group showed a trend towards worse left ventricle function: less shortening fraction (37.5 ± 3.2% vs. 40.7 ± 4.9%, *p* = 0.07), less ejection fraction (68 ± 4.4% vs. 71.2 ± 5.4%, *p* = 0.06) and a greater E/E’ ratio (6.4 ± 0.8 vs. 4.9 ± 1.04, *p* = 0.01).

## Discussion

Our results show that very preterm infants born in the post surfactant era have worse respiratory health, at least up to adolescence, than moderate and late preterm, as they required over twice as many hospital admissions due to respiratory problems and fourfold admissions to PICU. Adolescents born very preterm also showed higher asthma prevalence (22%), whereas the prevalence in moderately-late preterm (9.5%) was no greater than in the general population. In fact, being born before 32 wGA was, in our study, an independent risk factor for asthma at 13–14 years. Doyle et al.^34^, found similar prevalence of asthma (21%) in Australian adolescents 18 years old, born extremely preterm (< 28 wGA or < 1000 g) in the post surfactant era. However, their results are not totally comparable to ours, because their patients were older and the prevalence of asthma in their full-term control group was 19%, clearly superior to the prevalence in Spanish adolescents (9%)^28^. Hadchouel et al.^35^, also found prematurity and BPD to be associated with higher risk of asthma at 11 years in comparison with full-term birth. However, Jackson et al.^36^ in a cohort of children born extremely preterm (23 to 27 wGA) did not find association between BDP and asthma. This discrepancy with other authors may be explained by the extremely prematurity of Jackson´s patients; as Fierro et al.^37^ propose, the pulmonary consequences associated with prematurity should be seen as a spectrum of disease. BPD is one end of the barometer, but even without a diagnosis of BPD, patients born extremely premature have ongoing pulmonary morbidity. In our series BPD was associated with current asthma, although without reaching statistical significance, probably because there were only 21 patients with this diagnosis. Den Dekker et al.^38^, in a meta-analysis comprising data on 24,938 children born < 37 wGA, aged 3.9–19 years, found an odds ratio adjusted for asthma: 1.34 for preterm *vs*. full-term children. These results are also not comparable with ours since they compared all preterm children born < 37 wGA as a single group, without stratifying for gestational age, and again the control group was made up of full-term children. In a Finnish study population, the risk of asthma in children 0–19 years was 3.9-fold higher in those born < 32 wGA (17.8%) compared with full-term controls (5.5%). In late preterm, the prevalence of asthma was 9%. These data, although similar to ours, come from a group with a wide range of age, 0–19 years old. Our results confirm that early prematurity is an independent risk factor for asthma at 13–14 years with more than 2.5 times the risk associated with moderate and late prematurity. 

Although preterm birth has been associated with worse lung function compared preterm *versus* late and moderate preterm. Kotecha et al.^39^, in a study based on a heterogeneous sample of patients in terms of age (5–25 years) and time of birth (pre and post surfactant period), found lower values of lung function in preterm children. Hadchouel et al.^35^, in the EPIPAGE study found worse lung function at 11 years in 304 very preterm children (24–32 wGA) compared to 47 full-term children. In our study, as previously mentioned, we compared the lung function of the very preterm, not with full-term children, but with moderate and late preterm adolescents and it is crucial to take this into account when interpreting the results. In recent years several studies have highlighted the worse respiratory performance of moderate and late preterm children compared to those born at term^40^. Thunqvist et al.^41^, in a large prospective longitudinal birth cohort study, found that subjects born moderate to late preterm had lower lung function as measured by spirometry at 16 years of age compared with children born term. Similar results were reported by Kotecha et al.^42^, with significantly lower lung function at 8 to 9 years of age for children born after 33 to 34 weeks of gestation, compared with term control subjects. It is possible that the lack of difference in lung function values observed in our study can be explained by the fact that very preterm infants were compared to moderate and late preterm patients who, as mentioned above, have lower lung function values than full term children. But it should be also pointed out that all these preterm adolescents were born in the post surfactant era, with new therapeutic approaches, that probably have contributed to a better long-term functional evolution.

Regarding IUGR, defined in our study as fetal weight below 3 percentile or below 10 percentile and altered doppler of uterine, cerebral or umbilical arteries, was associated with lower spirometry values (FEV_1_ and FVC) in 13–14-year children with a history of premature birth in comparison with those appropriately in utero grown children born also preterm. And this association was independent of gender and gestational age. Greenough et al.^43^, in a follow-up study on 119 preterm infants, concluded that prematurely born infants who have suffered intra-uterine growth retardation could be at increased risk of impaired lung function at a median postnatal age of 10 months. Morsing et al.^44^ performed spirometry at median age 8.4 years (range 6.5–10.7) in 31 children born preterm with IUGR with a median gestational age 27 weeks. They found that lung function was reduced in the preterm-IUGR group at early school-age compared to controls born at term, but no differences were found when compared with preterm children without IUGR. More recently, Ronkainen et al.^13^ in a study on 88 preterm children matched with 88 term controls found that IUGR is an independent risk factor for lower lung function at 11 years. Our study, as well as Greenough et al.’s^43^ and Ronkainen et al.’s^13^, was not originally planned to investigate IUGR and the sample size was originally calculated for the difference between early preterm-born and moderate and late children, making subgroup analysis underpowered. This may explain why the association between IUGR and worse lung function was close to statistical significance, although not reaching it. On the other hand, as suggested by Ronkainen et al.^13^, over-representation of girls with IUGR does not weaken the observed difference between the IUGR and the no-IUGR groups since the association remained almost significant after adjustment for gender and prematurity. Anthropometric data also do not explain the differences in lung function between IUGR and no-IUGR since no differences in BMI, weight z-score and height z-score were detected among them at the time of the study. Therefore, our results suggest that, independently of prematurity, IUGR may have an additional influence on subsequent impaired lung function and its effect remains at least until the age of 13. The mechanisms that link IUGR with lung disease are not fully clarified. Recent studies demonstrated that IUGR inhibits alveolar formation early after birth as well as lung growth^45^. Also, experimental studies have shown that IUGR dysregulates growth factor signalling, activates inflammatory pathways and perturbs protease activity and thereby extracellular metabolism^46^. On the other hand, data from Nawabi et al. highlight that catch-up growth after IUGR activates myofibroblast function during late lung development, which in turn could favours lung growth or aggravates fibrotic processes and impairs thereby lung function later in life^47^.

Regarding arterial blood pressure (BP), the very preterm group showed significantly higher DBP, although all the values were normal for age. Extremely preterm (< 28 wGA) also showed significantly higher SBP and DBP than moderate and late adolescent preterm. Higher risk of hypertension has also been described in premature patients in adult life^2,48,49^, and could be explained by altered growth of vascular bed, decreased production of nitric oxide and diminished deposits of elastin, all of them associated with prematurity, that would contribute to an increased stiffness of vascular wall^1,40,50^. However, most studies have compared arterial blood pressure among preterm and full-term populations. Our results show than the frequency of higher blood pressure levels remains higher in very preterm adolescents when compared with not so extremely preterm controls. Although the difference compared with control subjects is on average only a few mm Hg, the risk induced may be significant as an increase of 5 mm Hg in blood pressure in a young adult has been shown to translate into a 34% higher risk of dying due to stroke later in life^51,52^.

Prematurity has been associated with changes in the morphology of both ventricles and systolic and diastolic dysfunction. Our results showed smaller size of the left ventricle, along with worse diastolic and overall function of both ventricles in very preterm children. In contrast, no differences were found between very premature and moderate and late preterm in length, basal diameter and mean diameter of right ventricle. Lewandowski et al.^50^ in 102 former preterm young adults, born in the pre-surfactant era, found smaller right ventricles but greater right ventricular mass. The difference with our findings, regarding the lower size of the right ventricle found by these authors, may be related with the peculiar morphology of this ventricle, that makes two-dimensional echocardiographic assessment less sensitive than MRI for detecting such alterations and with the milder course of our patients, born in the surfactant era.

Preterm adolescents with IUGR showed, in our study, poorer global cardiac function, with less shortening fraction, worse E/E’ ratio and longer MPI than non-IUGR preterm children. Several different patterns of cardiovascular remodelling in IUGR fetuses have been described, depending on the severity of the condition^50^, although cardiac remodelling and dysfunction in IUGR fetuses is mostly subclinical and requires sensitive method for identification^54^. Significant cardiac changes have also been described in IUGR neonates and infants up to 6 months of age^55^*.* Crispi et al., in a cohort of 80 IUGR and 120 controls evaluated at 5 years of age, reported a reduced stroke volume and subclinical longitudinal systolic and diastolic dysfunction in children with IUGR^56^*.* Recently, Sarvari et al., in 58 IUGR and 94 controls followed-up to 8–12 years, also found different cardiac shape with more spherical and smaller hearts and a decreased longitudinal motion and deformation and impaired relaxation in IUGR children^57^. Our results, along with those mentioned above, suggest that the effects of cardiovascular remodelling in IUGR- children persists up to adolescence (13–14 years). Since altered right ventricle function was also observed in very preterm children, this group of premature patients seems to be the most vulnerable to the remodelling changes associated with IUGR. Therefore, it is on these children that cardiovascular disease prevention programmes should be especially focused.

Estimated SPAP was higher in very preterm adolescent; even though this difference was not clinically significant, it may represent an increased vascular resistance that would persist into adolescence. It is necessary to bear in mind that SPAP is a dynamic parameter and, to obtain an accurate measurement, invasive techniques are needed.

When assessing left ventricle, no difference in systolic function was found, similar to Kowalski et al.^58^, findings. However, poorer diastolic and global function was detected when performing tissue doppler. The values obtained in the vast majority of our patients were considered to be normal, therefore we cannot conclude these changes are clinically significant. Nevertheless, this reduced function values may be the basis on which other risk factors, such as increased levels of fasting glucose and insulin, and elevated HOMA-IR, described in adults born prematurely, may contribute to higher risk of cardiovascular disease in adults born preterm^59^.

The main limitation of our study is the small number of BPD preterm adolescents in our sample, although our main goal was to evaluate the cardiac and lung function of premature children regardless of whether or not they had BDP.

In summary, our results show that, in the post surfactant era, prematurity < 32 wGA is an independent risk factor for asthma in adolescence, although is not associated with worse lung function in comparison with moderate and late preterm children. In contrast, IUGR is a risk factor for poorer lung function regardless of gestational age. Altered right ventricle function was observed in very preterm children and especially in IUGR ones. Our results suggest that IUGR in children born very preterm poses a greater risk for impaired cardiac and lung function in adolescence. Preventive measures should be encouraged in this high-risk population.

## Supplementary information

Supplementary Information.
