# Supplementary material for: Asthma prevalence, lung and cardiovascular function in adolescents born preterm
Source: Sci Rep. 2020 Nov 12;10:19616. doi: 10.1038/s41598-020-76614-0 (PMC7661536; doi:10.1038/s41598-020-76614-0)
Supplement: Supplementary file 1 — Supplementary Information. [file 41598_2020_76614_MOESM1_ESM.pdf]

SUPPLEMENTARY INFORMATION

Asthma prevalence, lung and cardiovascular function in adolescents born preterm

Maria Arroyas, PhD[1,2], Cristina Calvo, PhD [2,3,4,5], Santiago Rueda, PhD [6], Maria Esquivias, MD[6], Cristina Gonzalez-Menchen, PhD[6], Ersilia Gonzalez-Carrasco, PhD[1,2], Maria Luz Garcia-Garcia, PhD.[1,2,4]

1. Pediatrics Department. Severo Ochoa University Hospital. Leganes. Madrid. Spain
2. Alfonso X El Sabio University. Villanueva de la Cañada. Madrid. Spain
3. Pediatrics Department. La Paz University Hospital. Madrid. Spain
4. Translational Research Network in Pediatric Infectious Diseases (RITIP)
5. TEDDY Network (European Network of Excellence for Pediatric Clinical Research), Italy;
6. Pediatrics Department. Clinico San Carlos University Hospital. Madrid. Spain

Corresponding authors:

Maria Luz Garcia-Garcia

Pediatrics Department. Severo Ochoa University Hospital

Avenida Orellana s/n. Leganes 28911. Madrid. Spain

marialuz.hso@gmail.com

Maria Arroyas

Pediatrics Department. Severo Ochoa University Hospital

Avenida Orellana s/n. Leganes 28911. Madrid. Spain

mariarroyas@hotmail.com

Table 1S. Anthropometric Characteristics of the Study Groups at Follow-Up according to gestational age and the occurrence of intrauterine growth restriction (IUGR).

	<32 weeks N=74	≥32 weeks N=74	P value	IUGR N=20	Non-IUGR N=128	P value
**Height, cm***	158.0 (14.5)	162 (10.3)	0.007	158.0 (12.1)	161.4 (11.4)	0.125
**Height z-score***	-0.44 (-1.59)	-0.10 (1.20)	0.140	-0.53 (1.47)	-0.20 (1.34)	0.360
**Weight, kg***	48.3 (10.9)	55.3 (14.1)	<0.001	49.0 (13.3)	52 (16.2)	0.750
**Weight z-score***	-0.56 (0.92)	-0.08(1.14)	0.005	-0.38 (1.47)	-0.29 (1.32)	0.630
**Body mass index, kg/m²***	18.8 (4.3)	20.8 (5.6)	0.001	20.6 (6.1)	19.8 (5)	0.930
**Body mass index z-score***	-0.42 (1.09)	0.08 (1.54)	0.004	-0.13 (2.10)	-0.22 (1.39)	0.700

**Median (Interquartile range)*

Table 2 S. Affirmative answers in the ISAAC Questionnaire of respiratory symptoms of very preterm and moderately-late preterm adolescents.

**Answer**	**< 32 weeks N=74**	**≥ 32 weeks N=74**	**Odds Ratio (Confidence Interval 95%)**	**P value**
**1. Have you ever had wheezing episodes at any time?**	51 (68.9%)	43 (58.1%)	1.1 (0.9-1.4)	0.270
**2. Have you had wheezing episodes in the last 12 months?**	16 (21.6%)	7 (9.5%)	2.3 (1.1-5.2)	0.040
**3. How many wheezing episodes have you had in the last 12 months?**				
None	58 (78.3%)	68 (91.9%)		0.060
1-3	11 (14.9%)	6 (8.1%)		
4-12	4 (5.4%)	0		
>12	1 (1.4%)	0		
**4. How many times have you had symptoms in the night in the last 12 months?**				
Never	66 (89.2%)	72 (97.3%)		0.050
Less than once a week	8 (10.8%)	2 (2.7%)		
More than once a week	0	0		
**5. Wheezing episodes have you interrupted while speaking in the last 12 months?**	8 (10.8%)	4 (5.4%)	2 (0.6-6.3)	0.230
**6. Have you ever been diagnosed with asthma?**	30 (40.5%)	18 (24.3%)	1.7 (1-2.7)	0.050
**7. Have you had wheezing episodes while practicing sports?**	5 (6.8%)	5 (6.8%)	1 (0.3-3.3)	1
**8. Have you had dry cough in the night in the last 12 months?**	6(10.8%)	4 (5.4%)	2 (0.6-6.3)	0.230

Table 3S. Lung function measurements according to gestational age and the occurrence of intrauterine growth restriction (IUGR).

	**<32 weeks** N **=74**	**≥ 32 weeks** N **=74**	**P value**	**IUGR** N **=20**	**No-IUGR** N **=128**	**P value**
**FEV_1** (% predicted)*	94.8 ± 13.3	95.1 ± 13.9	p=0.890	88.7 ± 13.9	95.9 ± 13.3	0.027
**FEV_1 z-score****	-0.44 ± 1.13	-0.43 ± 1.17	p=0.950	-0.94 ± 1.17	-0.35 ± 1.12	0.034
**FVC** (% predicted)*	94.3 ± 13.6	94.7 ± 13.6	p=0.890	88.2 ± 13.6	95.5 ± 13.3	0.025
**FVC z-score****	-0.49 ± 1.15	-0.44 ± 1.18	p=0.810	-0.99 ± 1.17	-0.38 ± 1.14	0.028
**FEV_1/FVC** (% predicted)*	99.9 ± 9.1	100.1 ± 9.4	p=0.920	100.07 ± 8.5	100.04 ± 9.4	0.990
**FEV_1/FVC z-score****	0.16 ± 1.36	0.14 ± 1.42	p=0.920	0.11 ± 1.28	0.15 ± 1.4	0.890
**FEF_{25-75}** (% predicted)*	87.2 ± 21.5	94.1 ± 24.7	p=0.070	84.8 ± 22.3	91.5 ± 23.4	0.230
**FEF_{25-75} z-score****	-0.56 ± 1.18	-0.37 ± 1.14	p=0.330	-0.71 ± 1.12	-0.43 ± 1.17	0.300

Mean ± standard deviation
*Predicted values Zapletal.
**Z score Global Lung Initiative.
